# Accuracy Type Test for Rogowski Coils Subjected to Distorted Signals, Temperature, Humidity, and Position Variations

**DOI:** 10.3390/s22041397

**Published:** 2022-02-11

**Authors:** Alessandro Mingotti, Federica Costa, Lorenzo Peretto, Roberto Tinarelli

**Affiliations:** Department of Electrical, Electronic and Information Engineering, Guglielmo Marconi Alma Mater Studiorum, University of Bologna, Viale del Risorgimento 2, 40136 Bologna, Italy; federica.costa13@unibo.it (F.C.); lorenzo.peretto@unibo.it (L.P.); roberto.tinarelli3@unibo.it (R.T.)

**Keywords:** accuracy, type test, low-power instrument transformer, Rogowski coil, temperature, humidity, positioning, measurements

## Abstract

Low-Power Instrument Transformers (LPITs) are becoming the first choice for distributed measurement systems for medium voltage networks. However, there are still a lot of challenges related to their operation. Such challenges include their accuracy variation when several influence quantities are acting on them. Among the most significant influence quantities are temperature, electromagnetic field, humidity, etc. Another aspect that increases the importance of studying the LPITs’ accuracy behavior is that, once installed, they cannot be calibrated for several years; hence, one cannot compensate for in-field conditions. Hence, this work aims at introducing a simple type test for a specific LPIT, the Rogowski coil. First, an experimental setup to assess the effect of temperature, humidity, and positioning on the power quality accuracy performance of the Rogowski coil is described. Second, from the results and the experience of the authors it has been possible to design a specific type test. The test has the aim of finding the limits of the accuracy variations of a single Rogowski coil. Afterwards, such limits can be used to compensate for the in-field measurements, obtaining an overall higher accuracy. The results of this work may contribute to the always-evolving standardization work on LPITs.

## 1. Introduction

The concept of a smart grid would not exist without measurements. In fact, a smart grid is defined as an “electric power system that utilizes information exchange and control technologies, distributed computing and associated sensors and actuators, for purposes such as (i) to integrate the behavior and actions of the network users and other stakeholders, (ii) to efficiently deliver sustainable, economic and secure electricity supplies” [1]. Therefore, the collection and exchange of information is based on measurements. Such measurements are manipulated and converted by intelligent electronic devices (IEDs). However, the measurement stage is achieved by means of instrument transformers (ITs) and low-power instrument transformers (LPITs). LPITs are also referred to as nonconventional instrument transformers (NCITs) or simply sensors.

The choice of the IT also depends on the voltage level. For example, high voltage (HV) applications in the transmission network (TN) typically adopt legacy ITs [2,3]. This is mainly due to the need for consolidated reliability and to the significant availability of funds and space for the equipment installation. In other words, the LPIT technology is considered too “young” to be fully trusted by system operators (SOs). However, some encouraging HV applications with LPITs can be found in the literature [4,5].

Turning to the distribution side of the network (DN), the LPITs are becoming the main choice for several reasons. For example, they feature larger bandwidth, smaller size, lighter weight, reduced output, etc., compared to the ITs. Such characteristics, in addition to their cost-effectiveness, make the LPITs suitable for massive installation in the medium and low voltage sides (MV and LV) of the network. As a matter of fact, any expensive device could not spread among the node-crowded structure of the LV network.

In terms of standards, the work on ITs and LPITs is vivid. The reference standard series is the IEC 61869. It contains the IEC 61860-1 [6] and IEC 61860-6 [7], which are the general-purpose documents for ITs and LPITs, respectively. In addition, another 13 documents treat specifically each kind of transformer, such as IEC 61869-10 [8] and IEC 61860-11 [9], which deal with low-power current and voltage transformers (LPCTs and LPVTs), respectively. Of course, the documents relevant to ITs, such as IEC 61869-2 [10] and IEC 61860-3 [11], are the result of more years of studies than those for LPITs.

The focus of this work is on a widespread kind of LPCT; the Rogowski coil (RC). It is almost obvious that the peculiarities of such a device are suitable for several applications within, but not limited to, the power system scenario [12]. For example, they are used for fault detection in induction motors [13] and for measuring fast transients [14]. In [15], they were used as current sensors connected to the protections; while in [16], they were used for measuring high-frequency currents. It can be than concluded that the literature is full of studies on Rogowski coils, and new ones are published every day. With this work then; the authors want to extend the general knowledge on the accuracy performance of a Rogowski coil. This is completed by presenting a new and simple type test for assessing the accuracy performance of RCs when affected by several influence quantities. The type test originates from experimental measurements performed on off-the-shelf RCs and from previous literature (and experience). In particular, the experimental tests described in what follows include temperature, humidity, and position variation. Furthermore, the adopted current test signals were distorted, to reflect actual in-field measurements. The results were then evaluated in terms of the most adopted indices: ratio error and phase displacement.

Several papers related to the main topic of this work were found. For example, the single effect of distorted signals injected through the Rogowski coil was studied in [17,18]. In [19,20,21], instead, the effect of geometry on their performance was discussed and studied. As for [22,23], they dealt with the error evaluation and the effect of shielding on Rogowski coils, respectively. Finally, the authors in [24] started this research on Rogowski coils, analyzing the effect of positioning and temperature injecting ideal signals.

The added value of this work is the accuracy evaluation when the combination of the most influencing quantities with actual signals (typical in-field signals) is acting on RCs. The result is a complete guide, for manufacturers and Rogowski coil final users, in the form of a type test. Such a test can be performed to enhance the available information on the device accuracy. Note that the final user is guided in the design of their own test. Then, the information on the obtained accuracy can be used to correct in-field measurement results. This aspect is stressed in Section 2.

The remainder of this work is structured as follows: Section 2 describes the motivations for the need for new tests to be performed on Rogowski coils. This is achieved through discussion of the existing literature and relevant standards. In Section 3, the measurement setup to perform the experimental tests is described. The tests and their results are given in Section 4 and Section 5, respectively. Section 5 also contains the discussion of the results. Finally, Section 6 is the conclusion of the work with its achievements and the future directions for the research.

## 2. Importance of Testing

In this section the importance of testing is analyzed, whether for Rogowski coils or any device. The importance of calibrating equipment is, in fact, too often underestimated. To achieve accurate measurement results, it is not sufficient to buy and install a device. On the contrary, a full knowledge of the device performance should be achieved.

### 2.1. Calibration Process

From [1], the calibration is the “set of operations which establishes, by reference to standards, the relationship which exists, under specified conditions, between an indication and a result of a measurement”. The significance of such a process becomes clear if accurate measurement results are desired. However, if the calibration is quite a simple task for a single object, as in the case of a research lab, the same does not hold for a manufacturer. They deal with thousand devices per day/week; therefore, a calibration process should be simple, smart, and not time-consuming. In the ideal case, if each single device could be tested, there would be an increase in the knowledge about that device and its accuracy.

To support the importance of the calibration procedure, it is sufficient to analyze the amount of work in the literature. For example, [25,26] present a calibration system for ITs. In [27], the authors studied the calibration of direct current (DC) current transformers (CT); while in [28] it was conducted for digital ITs. A calibration procedure for legacy CTs was presented in [29], while different kinds of LPITs were calibrated in [30,31,32]. All of these works were extremely important for enhancing knowledge of ITs.

### 2.2. Modelling

The calibration (or characterization) of a device is often the first step for modeling it. Ideally, if the exact model of a device is known, any user could predict (hence, correct) the behavior of a device. The models are also used inside many software programs to simulate the power network and its operations. However, a mathematic approach alone is not sufficient to obtain a reliable model. In fact, it often happens that several unexpected non-idealities are introduced in the manufacturing process. Therefore, testing a device is always a recommended procedure.

In the literature, this is confirmed by many publications. For example, models for ITs, and, in particular, for CTs and voltage transformers (VTs), are developed in [33,34,35] respectively. As for LPITs, they have been modeled in [36,37] for LPVTs and Rogowski coils, respectively.

### 2.3. Periodical Calibration 

The final, and most critical, aspect to be considered before and during the installation of a device is its periodic assessment. In fact, there is no guideline in the IEC 61869 series about the time interval between the calibrations of an IT. Therefore, each manufacturer provides (even if not very often) information about the validity over time of their device’s accuracy. This typically ranges between 2 and 10 years, with peaks of 20 years in some cases. Note that the processes and methods that led to those timings are not regulated. Therefore, the final user is forced to trust the manufacturer’s experience. However, this could be avoided by including in the standards specific tests and/or recommendations on the periodic calibration of ITs.

Even the literature does not provide much information on this topic. However, researchers are sensitive to the issue, and they are working on on-site calibration for ITs. For example, on-site calibration systems have been developed for inductive VTs and CTs in [38,39,40,41,42]. In [43], instead, authors present an on-site calibration system for electronic ITs.

All in all, with this work, the authors want to introduce a complete type test, whose effectiveness has been experimentally validated. The test will provide information on the device accuracy in the most significant operating conditions. Furthermore, the test will guide the user on the test selection, depending on the target accuracy and/or the available testing equipment. Therefore, during the on-site operation of the device, it would be possible to adjust the measurement results and increase the overall accuracy.

## 3. Measurement Setup

**Figure 1:**
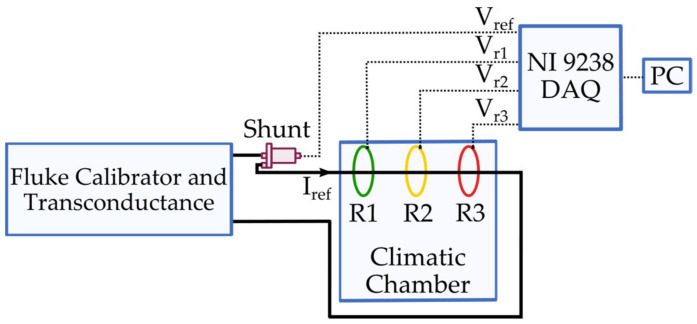
Measurement setup developed for the type test.

The measurement setup dedicated to the type test is depicted in Figure 1.

It consists of:

- A current source. The combination of the Fluke calibrator 6105 A and the transconductance 52120 A provides ideal or distorted currents. Such currents, referred to as I_ref_ in Figure 1, are injected into both the devices under tests (DUTs) and the reference measurement device. The manufacturer of the calibrator and the transconductance guarantees an accuracy performance of 0.009% of output error and 0.002% of range error for frequencies between 10 Hz and 850 Hz, 0.04% of output error and 0.004% of range error for frequencies between 850 Hz and 6 kHz.
- A reference measurement device. The measurements collected from the DUTs are compared with those of a resistive current shunt. The device, which has been fully characterized vs. temperature and vs. frequency, features an overall 0.01% of uncertainty. The shunt has a full scale of 100 A, and it consists of a 1 mΩ resistor. The output voltage of the shunt is referred to as V_ref_ (see Figure 1).
- A small note on the source and the reference: it is not strictly necessary to have both a reference source of current and a reference measurement device. Only one of them is sufficient to guarantee the traceability of the performed measurements. However, considering their availability, in this work both references have been used to ensure the accuracy level.
- A climatic chamber. It is a 550 L chamber, whose temperature can be varied between −40 °C and 180 °C (with an accuracy of 0.3 °C). Its relative humidity can be varied from 10% to 98% (with an accuracy between 1% and 3%) in the temperature range 10 °C to 95 °C. The chamber features two holes on the sides, which allow the user to insert power and communication cables.
- A set of three Rogowski coils (RCs). Each RC was made by a different manufacturer, but they all feature a 1% accuracy and a conversion ratio of 100 mV/1 kA. From now, they are referred to as R1, R2, and R3 for the sake of simplicity. The temperature ranges of the RCs are −20 °C to 70 °C, −40 °C to 80 °C, and −20 °C to 80 °C for R1, R2, and R3, respectively. Note, even the geometrical dimensions of the RCs are similar. They feature diameters between 8 cm and 10 cm. No other information is given by the manufacturers.
- A data acquisition (DAQ) system. The output voltage of the DUTs (V_r1_, V_r2_, and V_r3,_ for R1, R2, and R3, respectively) and V_ref_ are collected by using a NI9238. The DAQ has a full scale of ±500 mV, which is suitable considering the RCs conversion ratios. As for its accuracy, the NI9238 has a gain error of ±0.07% and offset error of ±0.005%. Finally, other interesting features of the DAQ are a 24-bit converter, 50 kSa/s/ch, and 1 GΩ input impedance. All the collected data are then computed via Labview software to obtain the desired parameters.

In brief, and explained further in Section 4, the desired distorted (or not) currents are injected through the DUTs and the reference shunt. Simultaneously, they are subjected to thermic, humidity, and position variations inside the chamber. Finally, the four acquired signals are manipulated to obtain the typical accuracy indices used to evaluate the Rogowski coils performance.

## 4. Experimental Tests

The design of the experimental tests was the result of a 3-step process. The first step consisted of selecting the temperature and relative humidity variations. The second step was used to fix the currents to be injected and measured by the DUTs. The third and last step had the aim of evaluating which positions of the DUTs were appropriated for the testing. Each step is discussed in the following subsections.

### 4.1. Temperature and Humidity

**Figure 2:**
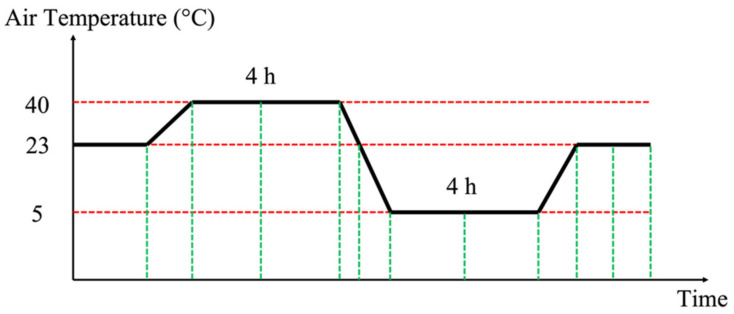
Accuracy vs. temperature test specified in [7].

For outdoor devices, temperature is probably one of the most significant influence quantities to be considered. All ITs are divided into three temperature categories. According to [6], these are −5/40, −20/40, and −40/40. The first number indicates the allowed minimum temperature, while the second number refers to the maximum. In [7], a new test for LPITs, to assess the accuracy vs. temperature, was added. The temperature cycle to be implemented is schematized in Figure 2.

In Figure 2, first, the minimum temperature was set according to the temperature class of the DUT. Second, the time interval between two temperature values and for a temperature plateau are given in terms of multiples of the DUT thermal constant. However, manufacturers do not always provide such information (as in the case of the DUTs tested in this work).

In light of the above, the selected temperature range was −5 °C to 40 °C, and the thermal constant was 1 h. The first choice was based on the average winter temperature in Italy, while the second choice was the result of experience in addition to the geometry of the DUTs. Therefore, as it can be seen in Figure 2, the temperature plateaus last for 4 h.

Turning to humidity, the decision is simpler considering that no indication is given in the IEC 61869 series. Therefore, what has been considered is to vary relative humidity between reasonable and practical values, 30%, 50%, and 80%.

Overall, the test involves a temperature cycle and three humidity values. In detail, the temperature cycle in Figure 2 is repeated for each humidity value.

### 4.2. Current Signals

The ambient conditions were fixed in Section 4.1. In this section, the test currents are described. Three current signals, referred to as H0, H1, and H2 were used. H0 is a 50 Hz sinusoidal signals without any kind of distortion. Signals H1 and H2 are identical to H0 but with a mix of superimposed harmonics. The description of the three signals is summarized in Table 1. Note that the percentage of the harmonics with respect to the 50 Hz component was selected as the maximum limit given in the Standard EN 50160 [44]. Furthermore, the rms value of the three signals was fixed at 80 A.

The choice behind the adoption of the signals H0, H1, and H2 was made considering the actual conditions at which the DUTs operate in field. Furthermore, signal H0 can be considered ideal; then, it represents a reference signal for further comparison. Signal H1 and H2, instead, have typical total harmonic distortion (THD) values according to the Standard IEEE 519 [45].

### 4.3. DUT Positioning

The last step of the test design process concerns the positioning of the RC. The need to fix the position originates from the fact that RCs are seldom centered with respect to the conductor they are mounted on. Among the reasons behind the uncentered positions, there is the (i) unavailability of centering systems or supports; and the (ii) lack of space in the installation cabinet (or secondary substation).

**Figure 3:**
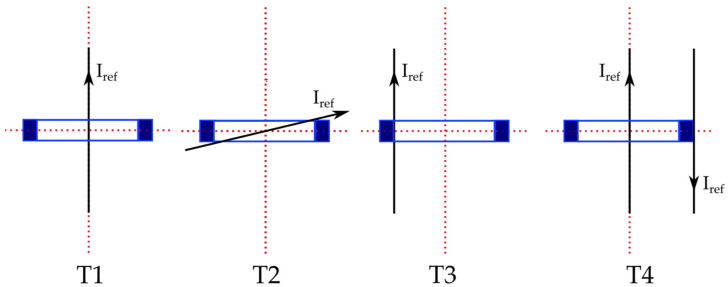
Testing positions for Rogowski coils according to [8].

Standard [8] include a set of positions in which the accuracy of the RCs needs to be tested. The four positions, referred to T1, T2, T3, and T4, are depicted in Figure 3.

Position T1 is the centered one, with the RC axes perpendicular to the conductor axis. Position T4 is identical to T1 but another conductor, carrying the same current as the main one, is close to the external surface of the RC. T4 can be also considered as the position that tests the effect of external magnetic fields on the performance of an RC.

Turning to positions T2 and T3, they are defined introducing the concept of positioning factor (PF). It is a parameter that ranges between 0 and 1, and it evaluates the angle between the conductor axis and the RC axis. For example, T1 has a PF = 0, while T3 has a PF = 1. As for T2, it has a 0 < PF < 1 to complete the possible cases. Finally, from the picture, it can be noted how the standard has defined a set of positions, which reflects the actual positions that an RC may assume in field.

All positions T1 to T4 were tested in this work.

### 4.4. Acquisition Details and Final Notes

Considering the details given in Section 4.1, Section 4.2 and Section 4.3 it can be summarized that (i) three current signals (H0, H1, and H2) were injected through R1, R2, and R3, while (ii) a temperature cycle was repeated three times with three relative humidity levels (30%, 50%, and 80%), and (iii) four positions were tested one after the other. The overall combination of the conditions resulted in 36 tests for each RC.

During each test:

One measurement was performed every 10 min.

Every measurement consisted of 100 repetitions of a 200 ms window.

For each repetition, the discrete Fourier transform (DFT) was applied on the acquired window (of the four signals), and the ratio error ε and phase displacement Δφ were computed as:

(1) $$\varepsilon =\frac{K_{r}U_{s}-I_{p}}{I_{P}}\times 100,$$

(2) $$\Delta \varphi =\varphi_{s}-\varphi_{p}.$$

The ratio error contains the nominal transformation ratio $K_{r}$ of the DUT, the rms voltage at the secondary terminals of the DUT ($U_{s}$), and the rms of the primary current flowing through the conductor ($I_{p}$). As for the phase displacement, it contains the phase-angle of the desired frequency component collected from the DUT ($\varphi_{s}$) and the primary conductor ($\varphi_{p}$), hence, from the reference shunt (V_ref_ signal).

Consequently, in each measurement the mean value of the 100 ε and 100 Δφ was computed and saved.

## 5. Experimental Results

This section begins with the results obtained from the tests described in Section 4. Then, the results are used to define the characteristic of a comprehensive type test to be performed on RCs.

### 5.1. Results for H0 and T1 tests

**Figure 4:**
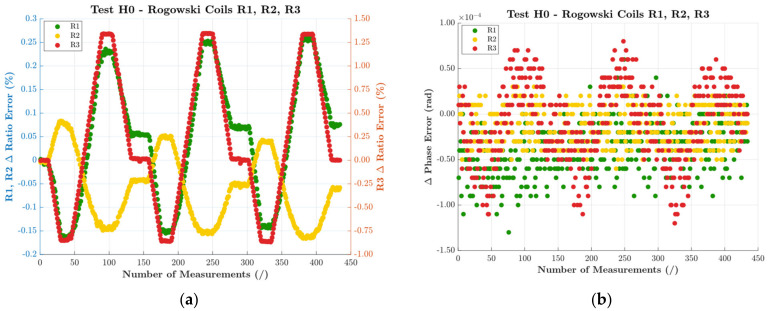
Results of the reference test performed with H0 and T1: (**a**) ratio error; (**b**) phase displacement.

The first set of results was obtained from the test that involves the temperature and humidity cycle, signal H0, and position T1. This test can be considered as the reference one for a clear understanding. For this purpose, Figure 4 depicts, for the three RCs, ε and Δφ variations in graphs (a) and (b), respectively. Note that the results are obtained subtracting each ε and Δφ from the first value obtained in the test (at 23 °C and in reference conditions). Furthermore, to increase ε readability, R1 and R2 are graphed in the primary vertical axis, while R3 is in the secondary axis (right hand side).

A first comment is on Δφ. For all RCs, its variation was quite limited compared to the accuracy classes. In fact, from Section 3, the given accuracy was 1%, hence, it may be associated to a 1 accuracy class, with limits 1% and 18 mrad for ε and Δφ, respectively. Another comment on Δφ is on the overall behavior; there was no significant effect due to the humidity variations. However, R3 behavior clearly followed the temperature cycles, highlighting a huge dependence on temperature (not reported for R1 and R2).

Turning to ε variations, the graph shows its dependency on the temperature. As a matter of fact, ε perfectly followed the temperature behavior for all RCs. What is different among the RCs under test was the sign of the variation. While R1 and R3 presented a negative variation with a temperature increase, R2 was coherent with the positive increase in the temperature. This result indicates that each device had a specific response to a temperature variation.

Finally, it can be observed from the graph that a change in the humidity value had a slight influence for R3, while the effect was more evident for R1 and R2. More details can be found in [46], which is dedicated to ideal testing on RCs.

Turning to the accuracy of the presented graphs (and the one of the following ones), ε and Δφ were measured with an accuracy of $10^{-4}$ (considering the% notation) and $10^{-8}$ rad, respectively. The values are standard deviation of the mean (100 values). Furthermore, they can be considered acceptable compared to the target uncertainty of the work.

### 5.2. Results for Positioning Tests T1 to T4

At this point it is interesting to understand the combined effect of temperature, humidity, and positioning on the accuracy performance of the DUTs when distorted signals are measured (H1 and H2). Note that the single effect of distorted signals has already been evaluated in [47]. 

**Figure 5:**
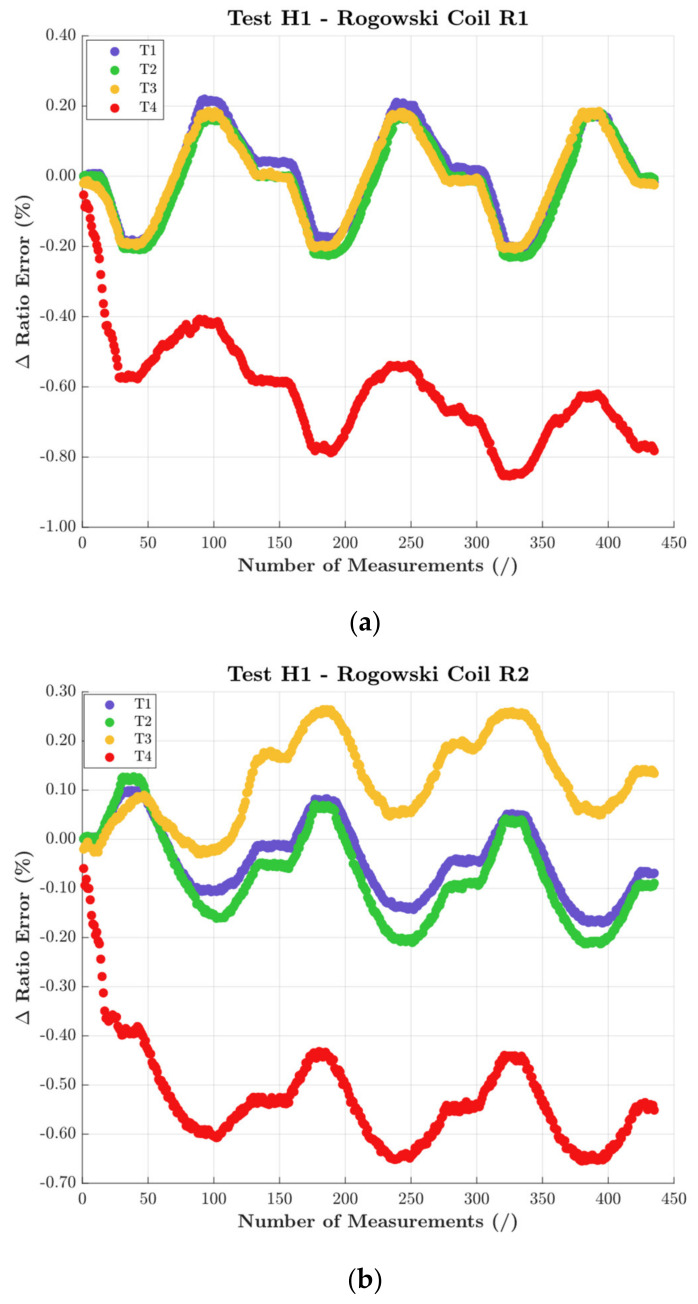

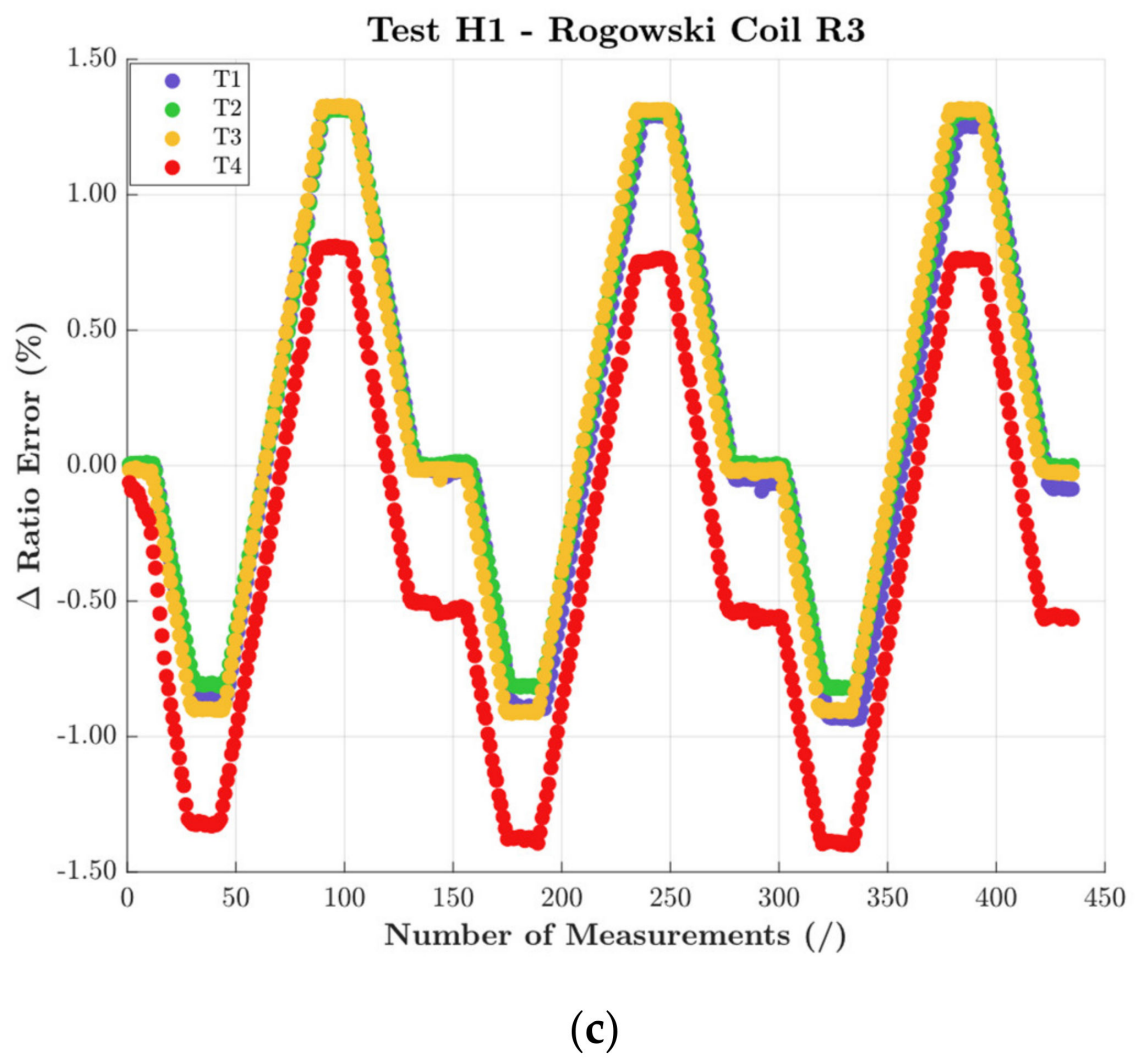
ε results of the test performed with H1 and all positions (**a**) R1, (**b**) R2, and (**c**) R3.

**Figure 6:**
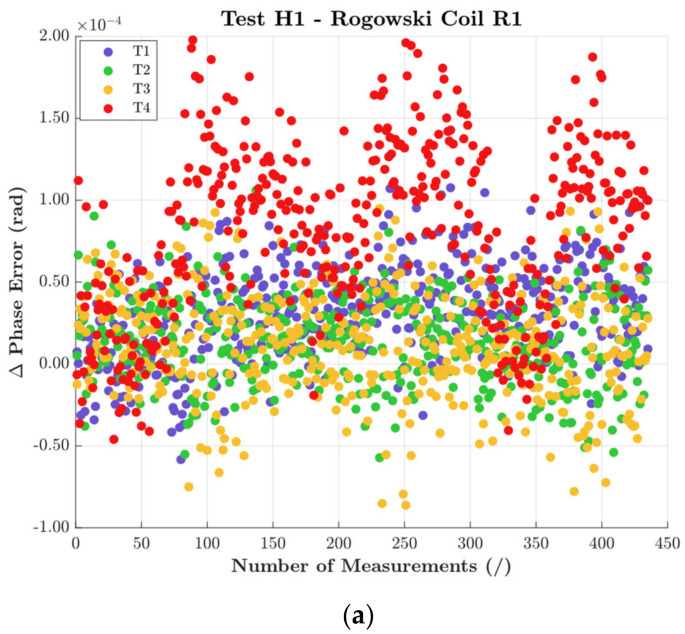

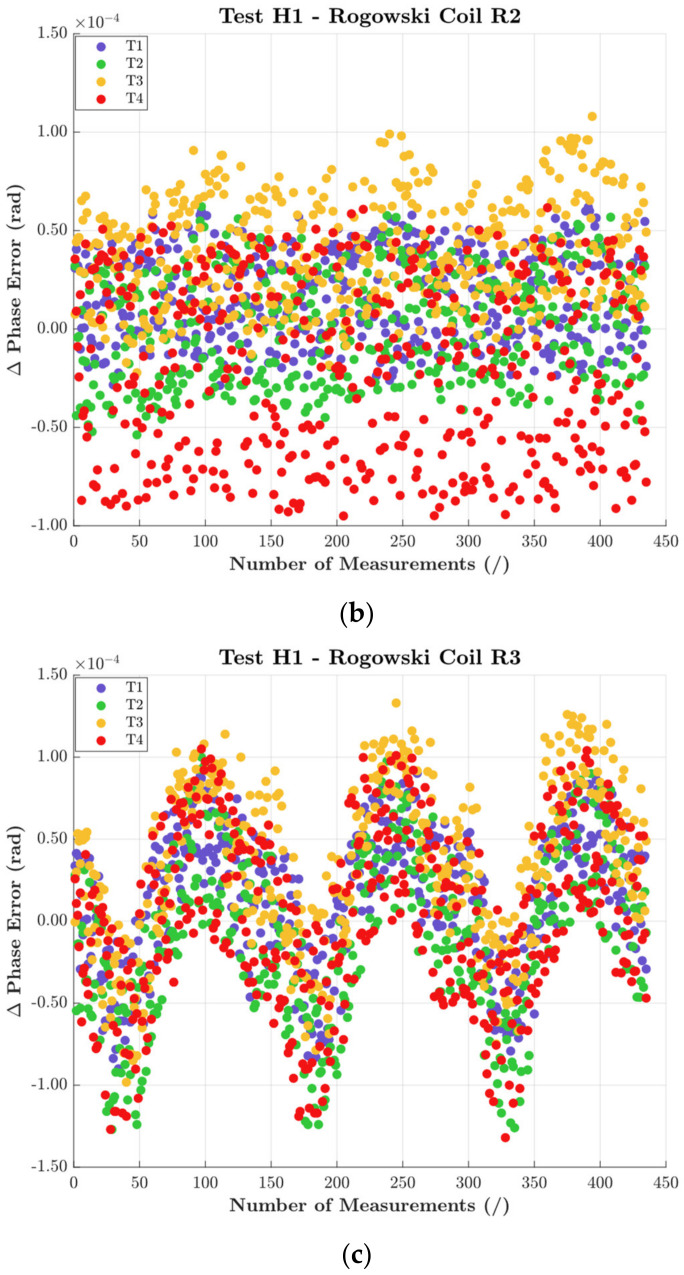
Δφ results of the test performed with H1 and all positions for (**a**) R1, (**b**) R2, and (**c**) R3.

Figure 5 and Figure 6 present ε and Δφ variations, respectively. In detail, Figure 5 includes graphs (a), (b), and (c), for R1, R2, and R3, respectively. Each graph depicts, with a different color, the four positions T1 to T4. Analogously, Figure 6 depicts the same graphs for Δφ.

Starting from Figure 5, the overall comment is that the accuracy of the DUTs is severally affected by their positioning. Furthermore, from the graph it can be understood what affected each single RC the most. In detail:

Position T4, which assesses the proximity effect, significantly affected all RCs, with variations of ε up to 1% (which is also the limit of the accuracy class).

Positions T2 and T3 did not significantly affect the accuracy of the three RCs. The only exception was R2, whose ε experienced a 0.3% variation with position T3. Such a position corresponds to a PF of 1 (the most severe).

R3 was overall more affected by temperature than by positioning. This emphasizes the need of testing each different RC.

The combination of positioning and humidity did not worsen the effect of the latter. 

Turning to Figure 6, it confirms the behavior described in Figure 4. R1 and R2 showed random variations of Δφ, while R3 presented a strong correlation with the temperature cycle. However, if such a correlation masks the effect of positioning on R3, this is not true for R1 and R2. Note in graphs (a) and (b) how position T4 influences Δφ. In fact, a positive and negative shift is observed for R1 and R2, respectively. These results confirm the influence of T4 on the accuracy of an RC.

A final comment is on the absolute value of the variations. Despite the above comments, in absolute terms, Δφ variations were almost negligible compared to the accuracy limits of the DUTs.

The same tests were performed, as previously described, with signal H2. The results, omitted here for the sake of brevity, presented coherent behaviors without any significant variation due to a different THD. However, more interesting comments on the harmonic evaluation are included in the following subsection.

### 5.3. Effect on the Harmonic Evaluation

Considering the increasing levels of pollution of the power network, it is more and more significant to assess the effect of distorted signals on the electrical assets. Therefore, this subsection focuses on the accuracy performance of the three RCs in terms of measured THD and harmonic components.

**Figure 7:**
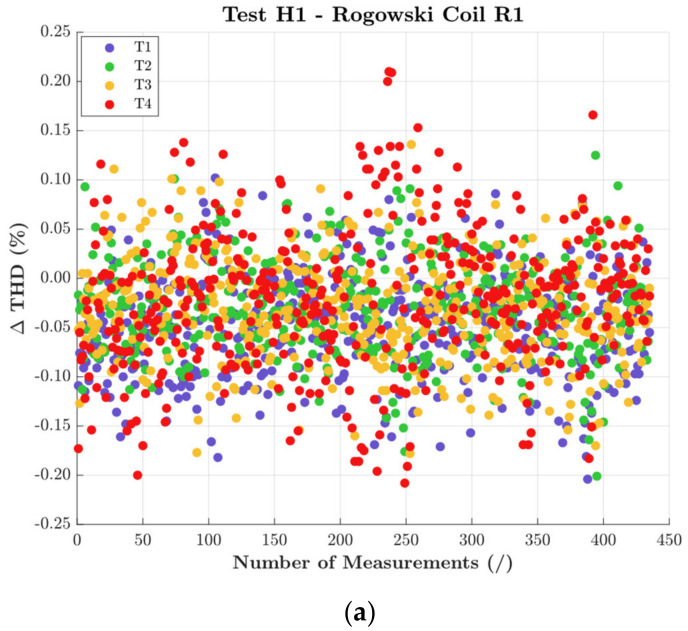

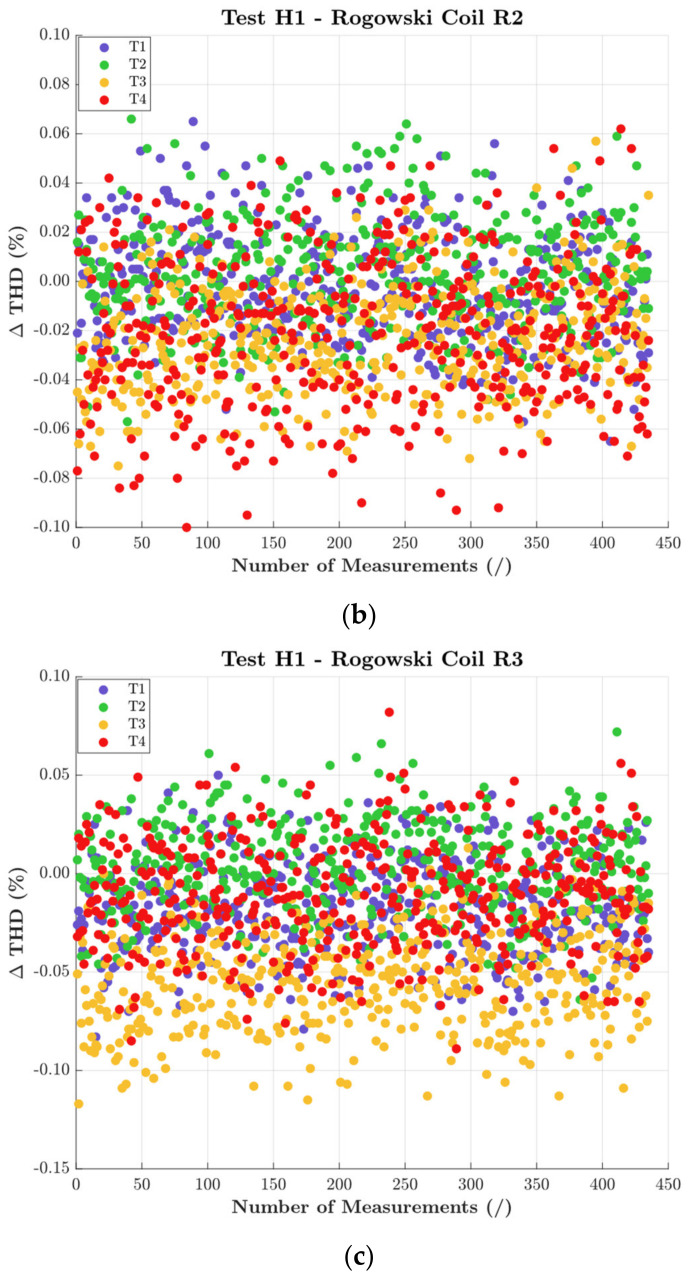
THD variations obtained with H1 and all positions, for (**a**) R1, (**b**) R2, and (**c**) R3.

The first set of results is given in Figure 7. For each RC, graphs (a), (b), and (c), respectively, the THD variation for all positions and during the temperature/humidity cycle is presented. The THD is plotted in terms of difference between the ideal value (e.g., 4.8% for H1) and the measured one.

The general comment is that THD was not affected either by positioning, temperature, nor humidity. Note, in fact, that the maximum recorded variations are in the order of 0.2% for R1. However, looking in more details the graphs, it can be observed that for R2 and R3 each position slightly moved the average value of the THD. This can be better perceived focusing on the yellow cloud of points obtained for R3 during the test with positioning T3.

**Figure 8:**
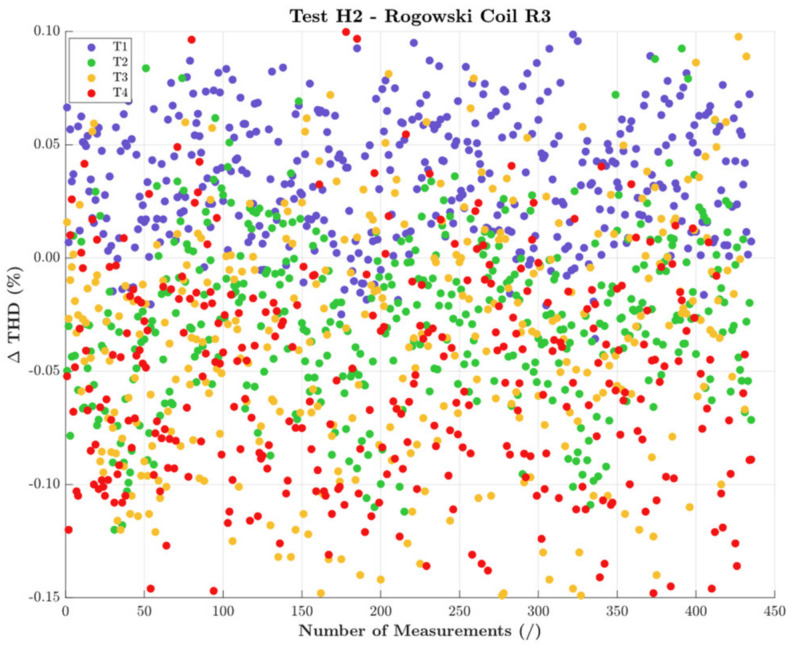
THD variations obtained with H2 and all positions for R3.

The same results were obtained from the tests with the signal H2. Therefore, for the sake of brevity, only the results of R3 are presented in Figure 8. This confirms that THD (a parameter that involves many quantities), and in particular its computation process, was not affected by the studied influence quantities.

**Figure 9:**
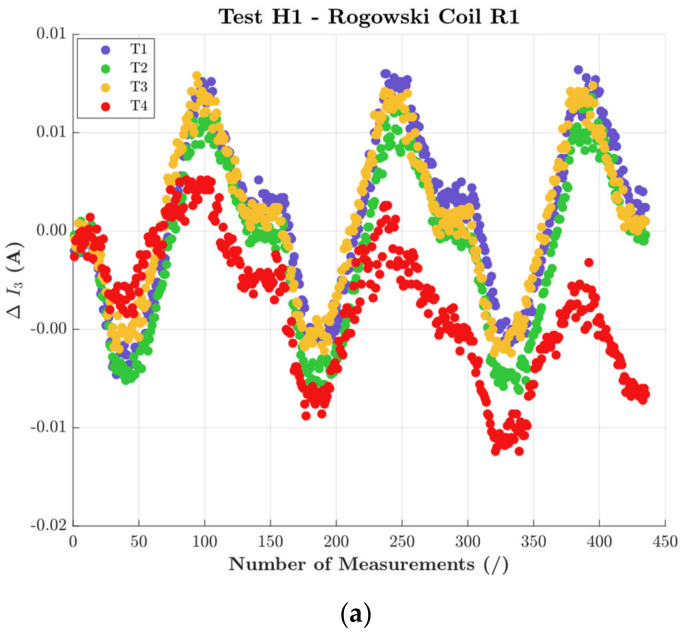

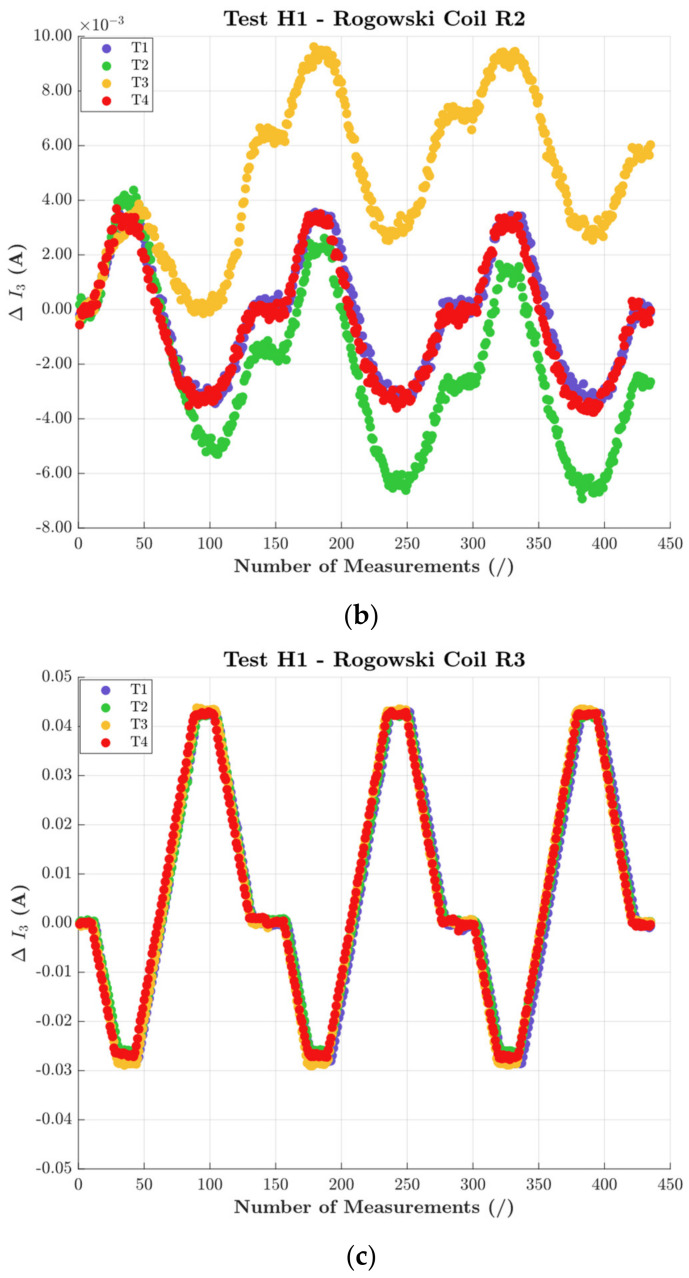
Third harmonic from tests with H1 and all positions, for (**a**) R1, (**b**) R2, and (**c**) R3.

The assessment of the harmonic evaluation proceeded with a focus on the single harmonics. To this purpose, Figure 9 shows the third harmonic (in ampere absolute value) extracted from the tests with signal H1 and positions T1 to T4 (hence, the same tests as Figure 7).

The first comment is a comparison with Figure 7 and Figure 8. Whereas from THD it is not possible to appreciate the presence of influence quantities affecting the RC, the opposite is true focusing on a single harmonic. Note the behavior of the third harmonic for all RCs strictly followed the temperature and humidity variations. Furthermore, the positioning affected R1 (T4) and R2 (T3), while R3 was insensitive to all positions.

Quantifying the variations, 10 mA, 10 mA, and 45 mA were observed for R1, R2, and R3, respectively.

**Figure 10:**
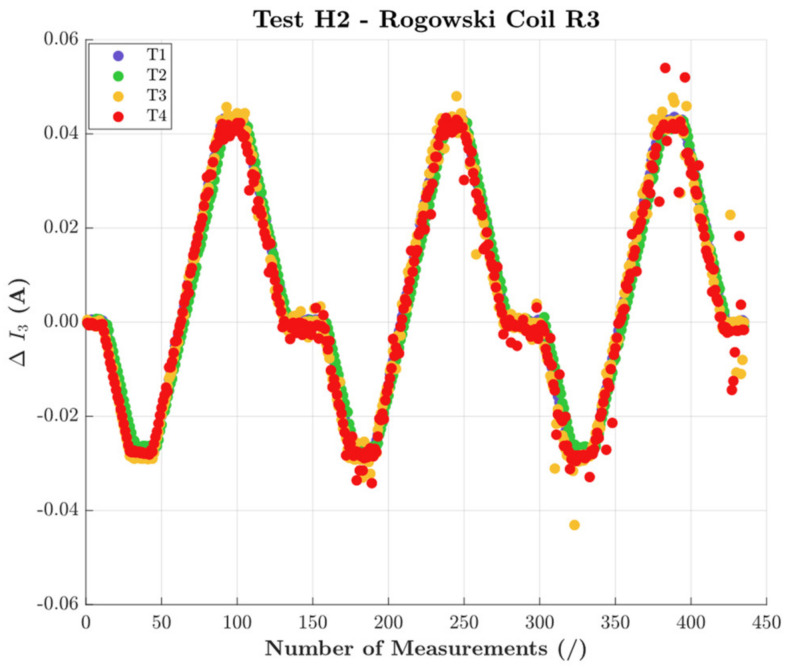
Third harmonic variations obtained with H2 and all positions for R3.

As for the tests with H2, the same results as in the case of H1 were obtained. Therefore, for the sake of brevity, the results for R3 are depicted in Figure 10.

### 5.4. Type Test Definition

The above test results show that the complete testing of an RC is a complex task. In detail, each RC has a different response to typical influence quantities that may affect its operation. The testing becomes even more challenging when more than one influence quantity is simultaneously affecting the device.

To this purpose, the aim of this subsection is to provide a guideline to perform a comprehensive type test to assess the accuracy performance of RCs. This test will allow the final user and the manufacturer to have a deeper knowledge of the device and to apply countermeasures when the off-nominal conditions happen.

**Figure 11:**
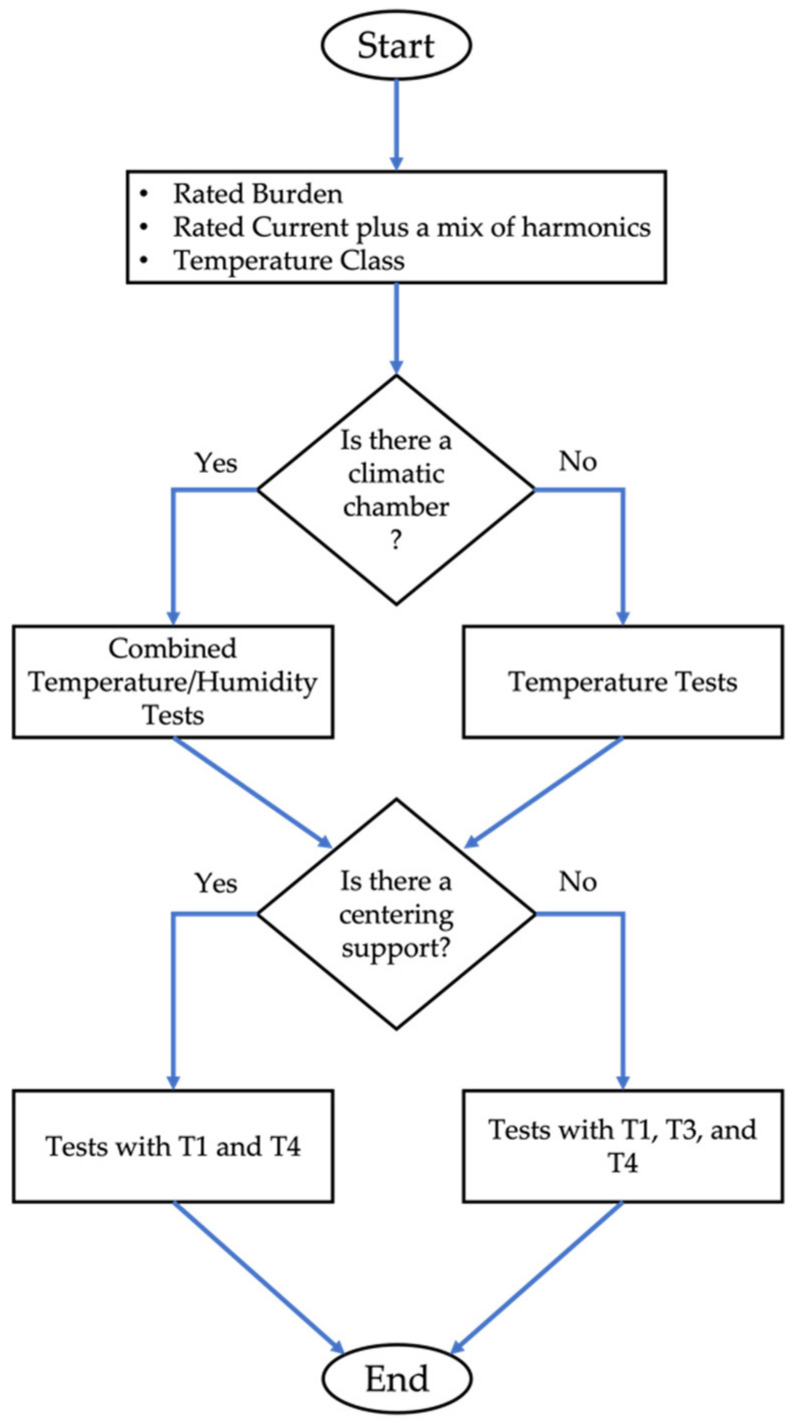
Type test design decision chart.

The type test design decision chart and the characteristics of the test are summarized in Figure 11. In detail:

For a type test, each product (LPCT) has its own rated values for current, burden, and temperature class. Note that the there is no rated current for RCs, just some recommended values. Otherwise, the rated current depends on the application. As for the mix of harmonic to superimpose to the main component, this can be conducted according to the standard [44]. However, the choice can be customized depending on the final application of the RC.

A manufacturer may have either a climatic or a thermostatic chamber to perform the tests. The tests over temperature are fundamental to evaluate the accuracy of the RCs. On the contrary, humidity variations have minor effects on the RC performance. Therefore, they could be used if a deeper knowledge is desired.

A critical aspect related to RCs is their positioning. However, a manufacturer may provide a centering device to fix the RC position once installed. Therefore, if that is the case it is sufficient to test the RC at the centered position T1 and at T4, which assesses the effect of magnetic fields. Otherwise, it is recommended to test also with T3, whose relevance has been demonstrated in this work.

Finally, all the tests provide ranges of variation and behaviors of ε and Δφ, which can be used to adjust the accuracy of each RC during its normal operation.

A final comment on the presented test is on the need for combined tests. It is not possible to rely on tests that assess one quantity at the time (as is the case in the actual version of the standard). On the contrary, there is the need to assess the instrumentation in as much a realistic condition as possible to simulate the in-field operations. For this purpose, the presented test may be helpful for the next version of the standards.

## 6. Conclusions

This paper presents a type test for Rogowski coils aimed at increasing the knowledge of their accuracy. The need for such a test comes from the in-field conditions in which the device operates. In fact, once installed, the Rogowski coil is typically subjected to more than one influence quantity that alters its accuracy. Therefore, even during its preliminary testing, the Rogowski coil should undergo actual tests with simultaneous application of several influence quantities. For this purpose, from the authors’ experience and from the results of this work, a specific type test was designed. Of course, it is a starting point for further standard versions. However, the test involves the main influence quantities that affect a Rogowski coil the most. Such quantities have been selected analyzing the performed experimental results. Finally, the aim of this test was to provide limits of variation for ε and Δφ of each type of Rogowski. After, such values can be used to adjust their in-field measured accuracy.

## Figures and Tables

**Table 1:** Characteristic of the Adopted Current Signals.

Signal	THD (%)	Description
H0	0	Pure 50 Hz signal
H1	4.8	H0 + 3rd, 11th, 17th, 23rd, and 35th
H2	9.2	H0 + H1 + all odd harmonics up to the 41st

## Data Availability

Not applicable.
